# Illumination of PRRSV Cytotoxic T Lymphocyte Epitopes by the Three-Dimensional Structure and Peptidome of Swine Lymphocyte Antigen Class I (SLA-I)

**DOI:** 10.3389/fimmu.2019.02995

**Published:** 2020-01-08

**Authors:** Xiaocheng Pan, Nianzhi Zhang, Xiaohui Wei, Yinan Jiang, Rong Chen, Qirun Li, Ruiying Liang, Lijie Zhang, Lizhen Ma, Chun Xia

**Affiliations:** ^1^Department of Microbiology and Immunology, College of Veterinary Medicine, China Agricultural University, Beijing, China; ^2^Institute of Animal Husbandry and Veterinary Science, Anhui Academy of Agricultural Science, Hefei, China; ^3^Key Laboratory of Animal Epidemiology of the Ministry of Agriculture, China Agricultural University, Beijing, China

**Keywords:** SLA, structure, CTL, epitope, PRRSV, vaccine

## Abstract

To investigate CTL epitope applications in swine, SLA-1^*^1502-restricted peptide epitopes matching porcine reproductive and respiratory syndrome virus (PRRSV) strains were explored by crystallography, biochemistry, and the specific pathogen-free (SPF) swine experiments. First, nine predicted PRRSV peptides were tested by assembly of the peptide-SLA-1^*^1502 (pSLA-1^*^1502) complexes, and the crystal structure of the SLA-1^*^1502 complex with one peptide (NSP9-TMP9) was determined. The NSP9-TMP9 peptide conformation presented by pSLA-1^*^1502 is different from that of the peptides presented by the known pSLA-1^*^0401 and pSLA-3^*^hs0202 complexes. Two consecutive Pro residues make the turn between P3 and P4 of NSP9-TMP9 much sharper. The D pocket of pSLA-1^*^1502 is unique and is important for peptide binding. Next, the potential SLA-1^*^1502-restricted peptide epitopes matching four typical genetic PRRSV strains were identified based on the peptide-binding motif of SLA-1^*^1502 determined by structural analysis and alanine scanning of the NSP9-TMP9 peptide. The tetrameric complex of SLA-1^*^1502 and NSP9-TMP9 was constructed and examined. Finally, taking NSP9-TMP9 as an example, the CTL immunogenicity of the identified PRRSV peptide epitope was evaluated. The SPF swine expressing the SLA-1^*^1502 alleles were divided into three groups: modified live vaccine (MLV), MLV+NSP9-TMP9, and the blank control group. NSP9-TMP9 was determined as a PRRSV CTL epitope with strong immunogenicity by flow cytometry and IFN-γ expression. Our study developed an integrated approach to identify SLA-I-restricted CTL epitopes from various important viruses and is helpful in designing and applying effective peptide-based vaccines for swine.

## Introduction

The development of new viral vaccines should be increasingly focused on biosafety, especially to remove the viral genetic material and to avoid the possibility of recombinant viruses developing due to the use of vaccines. In view of this central idea, diverse viral vaccines, such as cytotoxic T lymphocyte (CTL) and B cell epitope vaccines, have been experimentally researched in animals for the ongoing control of viral diseases and immunological deficiency diseases (1). Porcine reproductive and respiratory syndrome virus (PRRSV) is one of the most important swine pathogens and has caused significant economic losses in the swine industry worldwide for two decades (2). PRRSV is an enveloped positive-strand RNA virus with a viral genome of ~15 kb in length and contains 11 open reading frames (ORFs) (3). ORFs 1a and 1b are situated 5′-proximal to the polycistronic genome and encode two large non-structural replicase polyproteins, pp1a, and pp1b, which are processed into at least 14 non-structural proteins (nsps). Eight relatively small genes following ORF1 in the 5′ to 3′ direction encode four membrane-associated glycoproteins, three membrane proteins, and a nucleocapsid protein. Progress has been made in identifying nsp function related to RNA synthesis (nsp9 and nsp10), subgenomic mRNA synthesis regulation (nsp1), membrane-rearrangement (nsp2 and nsp3), replicative endonuclease (nsp11), major virulence factors (nsp3–8), and viral pathogenesis and host immunity (nsp1, nsp2, nsp4, nsp7, and nsp11) (4). The frequent mutation and recombination of the PRRSV RNA genome have resulted in the emergence of numerous variants (5, 6). These phenomena can cause the emergence of some virulent strains, such as the highly pathogenic PRRSVs that are causing enormous economic losses in Asia (7), and have led to the failure of vaccines against new emerging PRRSVs. There are considerable challenges and specific requirements in the development of novel vaccines to prevent PRRS, such as the CTL-epitope vaccine (8, 9).

The greatest challenge is that PRRSV can markedly suppress the swine immune defense system (10). The current evaluation of the PRRSV vaccine is based on its induced antibody response. Although high antibody titers can be produced after immunization, protection is not ideal because the key neutralizing antibodies (NAbs) against PRRSV appear late, typically >28 days post-infection (dpi), and usually at low levels (11). Furthermore, NAbs are usually specific for the homologous PRRSV strain and confer little cross-protection against heterologous strains (12, 13). Regarding CTL-mediated immunity, specific CTL responses have been observed in PRRSV (10, 14, 15). The virulent type 1 (Lena) PRRSV resulted in increased IL-1α production and a higher percentage of CD8+ T cells and IFNγ-producing cells compared with controls. Cross-reactivity against divergent PRRSV is also associated with cytotoxic CD8+IFNγ and CD8–IFNγ+ cells to a different extent (9). PRRSV-specific T cells could be observed as early as 2 weeks after infection, with the viral loads decreasing in persistent infection (13). Modified live vaccines (MLVs) could induce CTL immune responses and confer better protection against heterologous PRRSV strains than inactivated PRRS vaccines (16). These findings indicate that specific CD8^+^ CTL immunity may play an important role in controlling PRRSV infection. However, there is limited clear and direct evidence of CTLs eliminating PRRSV infection, and more basic immune reagents, such as the tetramer of swine major histocompatibility complex (MHC) class I with PRRSV peptide epitope, are required to address these important issues (9).

Swine MHC class I has been referred to as swine lymphocyte antigen (SLA-I). There are three classical SLA-I loci (SLA-1, SLA-2, and SLA-3) in the swine genome, and all are dominantly expressed (17). SLA-I molecules can present viral peptide epitopes to swine CD8^+^ T cells and induce the CTL response to kill the infected cells (18). Similar to human MHC (also known as human leukocyte antigen, HLA), SLA-I molecules are a highly polymorphic gene superfamily whose peptide-binding specificities are significantly influenced by highly variable sites (17). Thus, far, more than 100 SLA-I genes have been cloned (IPD; http://www.ebi.ac.uk/ipd/index.html), and two three-dimensional (3D) structures of peptide-SLA-I (p/SLA-I) molecules have been determined, revealing the peptide presentation characteristics of SLA-I molecules in swine (19, 20). Thus, the situation is favorable for the design and development of a novel viral CTL vaccine against swine PRRS based on these 3D structures of SLA-I molecules.

In an attempt to identify anti-PRRSV CTL epitopes in this study, first, predicted peptide epitopes derived from PRRSV were synthesized, and a trimolecular complex, the structure of the epitope from PRRSV-NSP9 (TMPPGFELY, termed NSP9-TMP9)-bound SLA-1^*^1502 (pSLA-1^*^1502), was solved. Next, the potential SLA-1^*^1502-restricted peptide epitopes matching four typical genetic PRRSV strains were identified. Finally, the immunogenicity of the CTL epitope was identified. Our results provide a novel strategy, i.e., the use of the MHC-restricted structural mechanism, to identify and validate CTL epitopes that could be used to develop a peptide-based vaccine against swine PRRS.

## Materials and Methods

### Prediction and Synthesis of PRRSV Peptides

Peptide epitopes were predicted by the NetMHCpan 4.0 Server (http://www.cbs.dtu.dk/services/NetMHCpan/) based on the whole protein sequences of four typical PRRSV strains (VR2332, GenBank accession no. EF536003.1; HB-13.9, GenBank accession no. EU360130.1; JXwn06, GenBank accession no. EF641008.1; and CHsx1401, GenBank accession No. KP861625.1). These potential non-apeptides were predicted using the SLA-1^*^1502 allele (GenBank accession no. HQ909439) and purified to >90% purity by analytical reverse-phase high-performance liquid chromatography (HPLC) (SciLight Biotechnology) (Table 1). These peptides were stored in lyophilized aliquots at −20 or −80°C after synthesis and were dissolved in dimethyl sulfoxide (DMSO) before use.

**Table 1:** Predicted peptides from PRRSV and influenza virus and their binding to SLA-1^*^1502 evaluated via *in vitro* refolding.

**Name**	**Sequence**	**Derived protein**	**Position**	**%Random^a^**	**Stability^b^**
PP1	SSSHLQLIY	PRRSV-GP5	34–41	0.388	++
PP2	IFLNCAFTF	PRRSV-M	48–56	0.473	++
PP3	LMLSSCLFY	PRRSV-GP4	96–104	0.592	++
PP4	IFLCCGFLY	PRRSV-GP3	9–17	0.538	++
PP5	SSAAAIPPY	PRRSV-NSP2	940–948	0.260	+
PP6	RWFAANLLY	PRRSV-NSP9	404–412	0.703	++
PP7	TMPPGFELY	PRRSV-NSP9	198–216	0.540	++
PP8	RTAIGTPVY	PRRSV-GP4	69–77	0.416	+
PP9	ISAVFQTYY	PRRSV-GP3	160–168	0.327	+
IP1	NSDTVGWSW	SI-NA	449–457	0.246	–

a *% Random is a base value for estimation of the binding affinities of peptides by the NetMHCpan 4.0 Server (http://www.cbs.dtu.dk/services/NetMHCpan/); the Rank threshold for strongly binding peptides is 0.100, and the rank threshold for weakly binding peptides is 1.000*.

b Stability is the capacity for peptide binding to SLA-1^*^1502. ++, peptide binds strongly and can tolerate anion-exchange chromatography; –, peptide does not bind SLA-1^*^1502; +, peptide binds SLA-1^*^1502 but cannot tolerate anion-exchange chromatography.

### Refolding of the SLA-1^*^1502 Complex

To assemble the pSLA-1^*^1502 complexes with each non-apeptide (Table 1), SLA-1^*^1502 heavy chain (HC) and swine β2m(sβ2m) inclusion bodies were refolded (in a 1:1:1 molar ratio) via the gradual dilution method we described previously (21, 22). The SLA-1^*^1502 HC and sβ2m inclusion bodies were also refolded without peptides or with non-combined peptides as negative controls. In addition, the SLA-1^*^0401 HC and sβ2m inclusion bodies were refolded with a positive peptide (amino acid sequence NSDTVGWSW) as a positive control. After 48 h of incubation at 4°C, the remaining soluble portion of the complex was concentrated and then purified via chromatography in a Superdex200 16/60 column, followed by Resource-Q anion-exchange chromatography (GE Healthcare), as previously described (21).

### Crystallization and Data Collection of pSLA-1^*^1502

The purified complex (44 kDa) of pSLA-1^*^1502 with the NSP9-TMP9 peptide (amino acid sequence TMPPGFELY, derived from residues 198–206 of the PRRSV non-structural protein) was dialyzed against crystallization buffer (20 mM Tris–HCl pH 8.0, 50 mM NaCl) and concentrated to 12 mg/mL. The sample was then mixed with reservoir buffer at a 1:1 ratio and crystallized via the hanging-drop vapor diffusion technique at 277 and 291 K. Index Kits (Hampton Research, Riverside, CA) were employed to screen the crystals. With a protein concentration of 12 mg/mL, crystals of pSLA-1^*^1502 were obtained in 10–14 days from index solution No. 65 (0.1 M Bis-Tris pH 5.5, 0.1 M ammonium acetate, 17% PEG 10 000) at 4°C. Diffraction data were collected at a resolution of 2.2 Å (pSLA-1^*^1502) with an in-house X-ray source (Rigaku Micro-Max007 desktop rotating anode X-ray generator with a Cu target operated at 40 kV and 30 mA) and an R-AXIS IV^++^ imaging plate detector at a wavelength of 1.5418 Å. The crystals were first soaked in reservoir solution containing 25% glycerol as a cryoprotectant and then flash-cooled in a stream of gaseous nitrogen at −173°C (23). The collected intensities were indexed, integrated, corrected for absorption, scaled, and merged by using the HKL2000 package (24).

### Structural Determination and Refinement of pSLA-1^*^1502

The structures of pSLA-1^*^1502 with NSP9-TMP9 were solved via molecular replacement using the MOLREP program with HLA-A^*^1101 (PDB code, 1Q94) as the search model. Extensive model building was performed by hand with COOT (25), and restrained refinement was performed with REFMAC5. Additional rounds of refinement were conducted by using the phenix.refine program implemented in the PHENIX package (26) with isotropic atomic displacement parameter (ADP) refinement and bulk solvent modeling. The stereochemical quality of the final model was assessed with the PROCHECK program (27). Data collection and refinement statistics are listed in Table 2.

**Table 2:** X-ray diffraction data processing and refinement statistics.

**Parameter**	SLA-1^*^1502-NSP9-TMP9
**Data processing**	
Space group	P2_1_2_1_2_1_
Unit cell parameters (Å)	a = 66.058, b = 74.059, c = 98.596 α = 90.00, β = 90.00, γ = 90.00
Resolution range (Å)	50.00-2.20 (2.20–2.28)^a^
Total reflections	197,524
Unique reflections	24,678
Avg redundancy	7.9 (7.9)
Completeness (%)	99.5 (98.9)
*R*_merge_ (%)^b^	8.3 (28.6)
Avg *I*/σ (*I*)	27.366 (7.517)
**Refinement**	
Resolution (Å)	29.607-2.199
*R*_factor_ (%)^c^	20.0
*R*_free_ (%)	24.3
**R M S Deviations**	
Bonds (Å)	0.014
Angles (°)	1.140
Average B factor	26.602
**Ramachandran plot quality**	
Most favored region (%)	91.5
Allowed region (%)	8.5
Disallowed region (%)	0.0

a *Values in parentheses are for the highest-resolution shell*.

b *R_merge_ = Σ_hkl_Σ_i_|I_i_(hkl) – <I(hkl>|/Σ_hkl_Σ_i_ I_i_(hkl), where I_i_(hkl) is the observed intensity and <I(hkl)> is the average intensity from multiple measurements*.

c *R = Σ_hkl_|| F_obs_ | – k | Fcalc | |Σ_hkl_| F_obs_|, where R_free_ is calculated for a random chosen 5% of reflections, and R_work_ is calculated for the remaining 95% of reflections employed for structural refinement*.

### Determination of the Circular Dichroism Spectra and Thermal Unfolding of pSLA-1^*^1502

The thermostability of SLA-1^*^1502 with six mutant peptides was examined via circular dichroism (CD) spectroscopy. CD spectra were measured at 20°C in a Jasco J-810 spectropolarimeter equipped with a water-circulating cell holder. Far-UV CD spectra (180–260 nm) were collected at a protein concentration of 0.2 mg/ml in 20 mM Tris (pH 8.0) buffer in a cuvette with a length of 1 mm at 0.1-nm spectral resolution. The ellipticity at 218 nm was continuously recorded during heating. Thermal denaturation curves were obtained by monitoring the CD value at 218 nm in a cell with an optical path length of 1 mm as the temperature was raised from 25 to 90°C at a rate of 1°C/min. The temperature of the sample solution was directly measured with a thermistor. The fraction of unfolded protein was calculated from the mean residue ellipticity (θ) by the standard method: the unfolded fraction (%) is expressed as (θ–θ_N_)/(θ_U_-θ_N_), where θ_N_ and θ_U_ are the mean residue ellipticity values in the fully folded and fully unfolded states. The midpoint transition temperature (Tm) was determined by fitting the data to the denaturation curves by using the Origin 8.0 program (OriginLab), as described previously (28).

### Tetramer Preparation

The tetrameric pSLA-1^*^1502 complex was constructed according to a previously described method (29). Briefly, a sequence containing a BirA enzymatic biotinylation site was added to the C-terminus of the SLA-1^*^1502 HC via PCR. The PCR primers and conditions were as described previously (30). Then, the entire construct was cloned into the pET-21a(+) vector, which was subsequently transfected into *Escherichia coli* strain BL21(DE3) for protein expression. The inclusion bodies of recombinant SLA-1^*^1502 HC containing the BirA site and of sβ2m were refolded with the NSP9-TMP9 peptide as described above. The pSLA-1^*^1502 complex was then purified and biotinylated by using the BirA enzyme (Avidity Aurora, CO). Finally, the complex was purified and tetramerized by mixing pSLA-1^*^1502-BSP with PE-labeled streptavidin (BioSource International, Camarillo, CA) at a molar ratio of 4:1, after which the samples were separated by using 100 KDa Millipore tubes. SDS-PAGE electrophoresis was used to determine the efficiency of tetramerization.

### Evaluation of the Immunogenicity of NSP9-TMP9 in Swine

A total of nine specific pathogen-free (SPF) swine (15 kg, 8–9 weeks old). Beijing Center of SPF Swine Breeding and Management) expressing the SLA-1^*^1502 alleles were divided into three groups: MLV, MLV+NSP9-TMP9, and a blank control group. For initial immunization, the MLV and MLV+NSP9-TMP9 groups were injected with an attenuated PRRSV vaccine according to the manufacturer's instructions (Boehringer-Ingelheim, Ingelvac). After seven days, for the second immunization, the MLV + NSP9-TMP9 group was injected with the NSP9-TMP9 peptide mixed with complete Freund's adjuvant (CFA, 1:3 emulsification). The MLV group was injected with the MLV peptide mixed with CFA. Seven days later, peptide mixed with incomplete Freund's adjuvant (IFA, 1:3 emulsification) was injected into the MLV+NSP9-TMP9 group. The MLV group was injected with MLV mixed with IFA. The immune dose of the peptide was 0.1 mg/kg body weight. The control group was injected with phosphate-buffered saline (PBS), deionized water mixed with CFA (1:3 emulsification), and deionized water mixed with IFA (1:3 emulsification) at the same time as the immunization group. Equivalent volumes were used in the immunization group and the control group. Blood was collected from the anterior vena cava, and peripheral blood mononuclear cells (PBMCs) were isolated by the kit according to the manufacturer's instructions (Solarbio). The PBMCs were incubated for 30 min at 37°C in staining buffer (PBS with 0.1% BSA and 0.1% sodium azide) containing the PE-labeled tetrameric complex and the FITC-labeled anti-CD8 monoclonal antibody. The cells were then washed once with staining buffer and detected via flow cytometry. More than 10^6^ cell events were acquired for each sample. Cells stained with PE-labeled tetramers and a FITC-labeled anti-CD8 monoclonal antibody were counted as CTL response cells (31). The results for fluorescence-activated cell sorting (FACS) data are presented as the mean ± standard error of the mean (SEM) for the three animals in each group. Statistical analysis was performed using GraphPad Prism 7 (https://www.graphpad.com) for Windows. Significant differences (*P* < 0.01) between means were tested by two-tailed Student's *t*-test.

### Production of IFN-γ in Swine After Immunization With NSP9-TMP9

One week after immunization with NSP9-TMP9 or deionized water, PBMCs were collected from immunized and control groups. These PBMCs were stimulated with NSP9-TMP9 peptide at a concentration of 2 μg/ml. PHA was added at the same concentration to each positive control group, while an equivalent volume of PBS was added to the negative groups. Swine IFN-γ in the supernatant was detected via an ELISA kit according to the manufacturer's instructions (Invitrogen) after the cells had been incubated at 37°C for 18 h.

## Results

### PRRSV Peptide Prediction and Verification of SLA-1^*^1502

Six SLA-I alleles were cloned from Landrace pigs. SLA-1^*^1502 showed better PRRSV peptide-binding ability than the others (Table S1) according to *in silico* prediction (http://www.cbs.dtu.dk/services/NetMHCpan). Nine PRRSV peptides, all of which could be presented by SLA-1^*^1502, were synthesized to test this prediction (Table 1). All nine peptides could form complexes with SLA-1^*^1502 and swine β2m (pSLA-1^*^1502) by *in vitro* refolding. The stable pSLA-1^*^1502 complexes were further used to screen the crystal structures.

### 3D Structure of pSLA-1^*^1502

**Figure 1:**
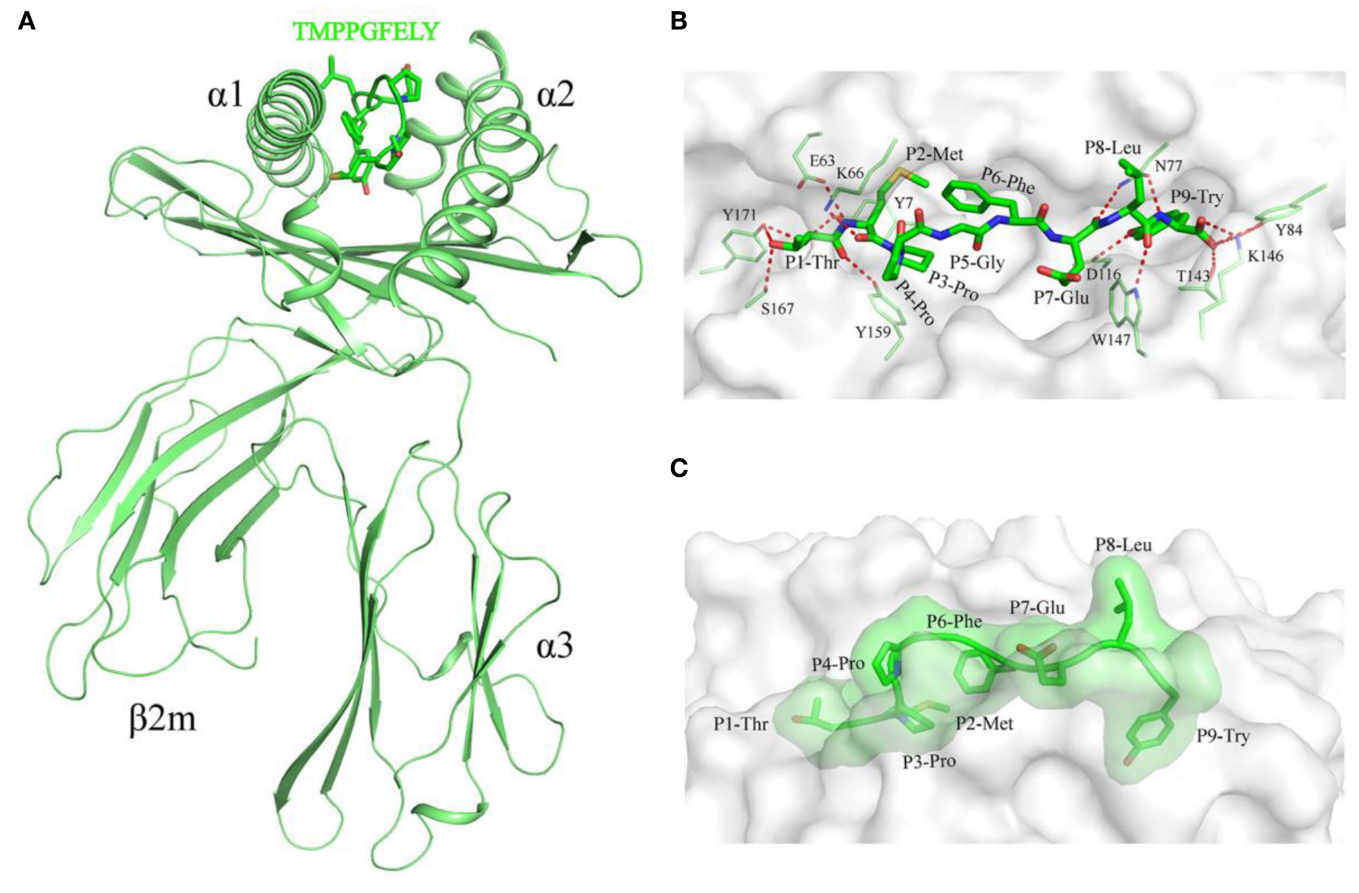
Structural overview and NSP9-TMP9 peptide presentation in pSLA-1*1502. **(A)** Overall structure of SLA-1*1502 in complex with the NSP9-TMP9 peptide. The non-apeptide is shown in stick representation between the α1 domain and α2 domain of pSLA-1*1502. **(B)** Interactions between the NSP9-TMP9 peptide and residues in the PBG. The hydrogen bonds are represented as red dashes. **(C)** Surface model of NSP9-TMP9. Most regions of P4-Pro and P8-Leu are located outside the PBG.

SLA-1^*^1502 in complex with NSP9-TMP9 was crystallized in the P2_1_2_1_2_1_ space group with a high resolution of 2.20 Å (Table 2). One asymmetric unit contains only one SLA-1^*^1502 molecule. The pSLA-1^*^1502 complex displays a canonical p/MHC I structure, including the α1, α2, and α3 domains of the HC and the light chain sβ2m. NSP9-TMP9 is located in the peptide-binding groove (PBG) formed by the α1 and α2 domains (Figure 1A). The root mean square differences (RMSDs) between SLA-1^*^1502 and two other solved p/SLA I structures (SLA-1^*^0401, PDB code: 3QQ3; SLA-3^*^hs0202, PDB code: 5H94) were found to be 0.446 and 0.592, respectively, indicating similarities among the overall structures of the p/SLA I molecules. The NSP9-TMP9 peptide is fixed by 15 hydrogen bonds with residues in the N- and C-termini of the PBG, and no hydrogen bonds were observed in the middle portion (P3–P7) (Figure 1B). Based on the surface model, the P4 and P8 residues are located outside the PBG, and their side chains are solvent accessible, especially the P8 residue, which is at the top position of the NSP9-TMP9 peptide conformation (Figure 1C).

### Pocket Composition of pSLA-1^*^1502 Bound to NSP9-TMP9 Peptide

**Figure 2:**
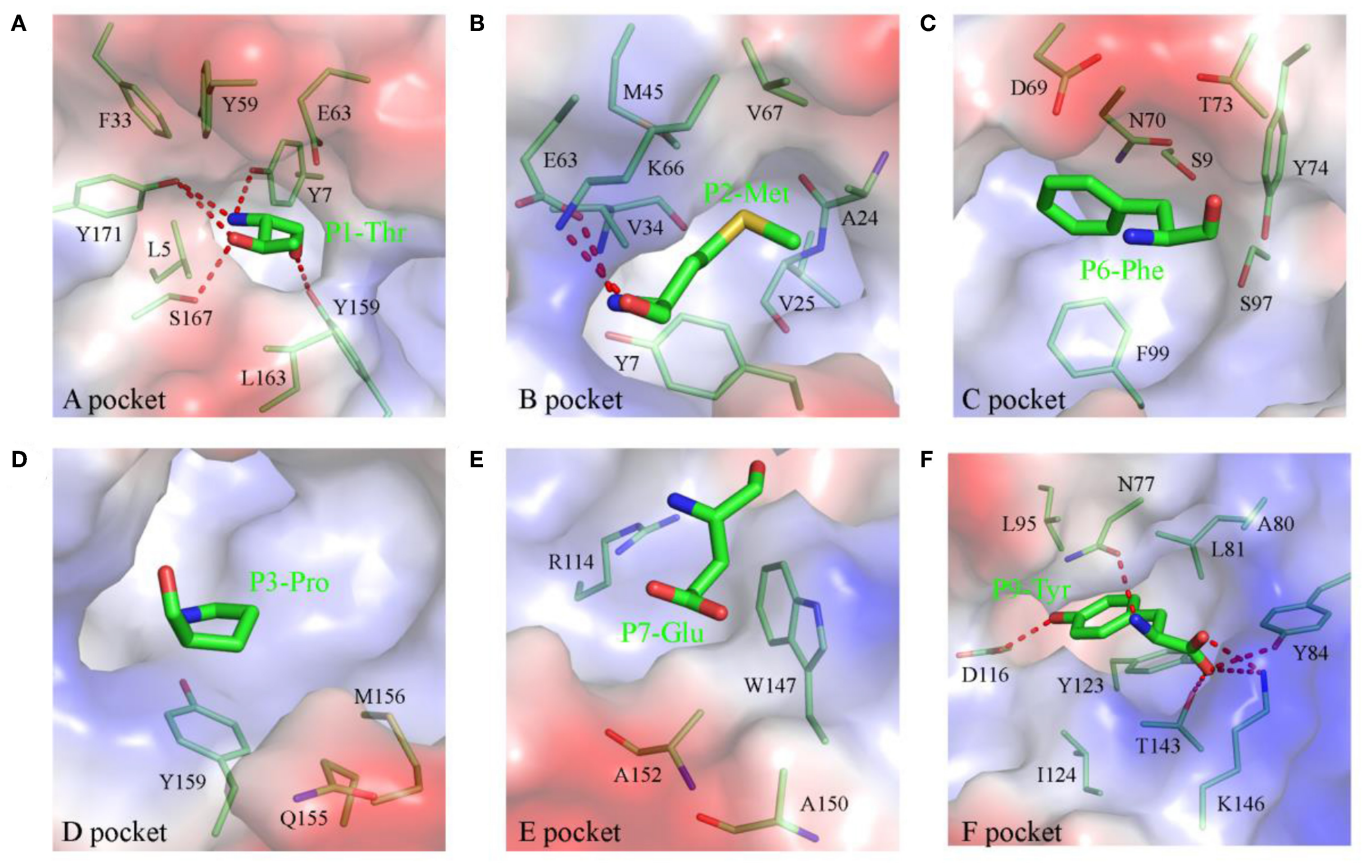
Composition and polarities of the six pockets of pSLA-1*1502 bound to NSP9-TMP9 peptide. The pockets are shown in surface representation with their polarities colored as follows: red, negatively charged; white, non-polar; and blue, positively charged. The residues forming these pockets (light green) and the bound peptide (C, green; N, blue; O, red) are labeled. The hydrogen bonds between peptides and the red complex are shown with dashes. **(A)** Pocket A with residue P1 (Thr). **(B)** Pocket B with residue P2 (Met). **(C)** Pocket C with residue P6 (Phe). **(D)** Pocket D with residue P3 (Pro). **(E)** Pocket E with residue P7 (Glu). **(F)** Pocket F with residue P9 (Tyr).

**Figure 3:**
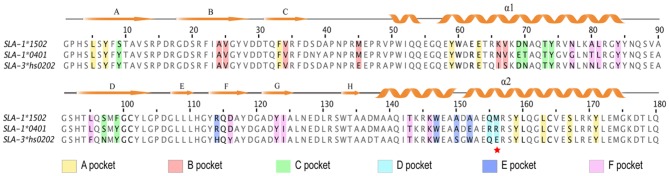
Structure-based amino acid sequence alignment of SLA-1*1502, SLA-1*0401, and SLA-3*hs0202 alleles. The amino acid sequences of the PBGs of SLA-1*1502, SLA-1*0401, and SLA-3*hs0202 were aligned, and the secondary structural elements are indicated above the sequences. Orange arrows indicate β-strands, and orange cylinders denote α-helices. The residues in pockets A-F are highlighted with different colors. The residues at position 156 in the D pocket are indicated with a red star.

The compositions and polarities of the six pockets of pSLA-1^*^1502 are shown in Figure 2, and the interactions between the NSP9-TMP9 peptide and these pockets are listed in Table 3. The pockets of pSLA-1^*^1502, p/SLA-1^*^0401, and p/SLA-3^*^hs0202 are compared in Figure 3. The A pocket of pSLA-1^*^1502, composed of Leu^5^, Tyr^7^, Phe^33^, Tyr^59^, Glu^63^, Tyr^159^, Leu^163^, Ser^167^, and Tyr^171^, fixes P1-Thr via hydrogen bonds and strong van der Waals forces (VDWs) (Figure 2A; Table 3). The residues forming the A pockets of SLA I molecules, including Ser^167^, are highly conserved (Figure 3). In most MHC I molecules of other species, the residue at position 167 is Trp (32). Due to the small Ser^167^ residue, the N-terminus of the PBG of SLA I molecules appears to be more open than in other MHC I molecules.

**Table 3:** Hydrogen bonds and van der Waals interactions between the NSP9-TMP9 peptides and complexes.

**Complex**	**Peptide**		**Hydrogen bond partner**		**Van der Waals contact residues^**a**^**
	**Residue**	**Atom**	**Residue**	**Atom**	
SLA-1^*^1502 /sβ2m/TY9	P1-Thr	N	Tyr^7^	OH	Leu^5^, Tyr^7^, Phe^33^, Tyr^59^, Glu^63^, Tyr^159^, Leu^163^, Ser^167^, Tyr^171^
			Tyr^171^	OH	
		O	Tyr^159^	OH	
		OG1	Ser^167^	OG	
			Tyr^171^	OH	
	P2-Met	N	Glu^63^	OE1	Tyr^7^, Ala^24^, Val^25^, Val^34^, Met^45^, Glu^63^, Lys^66^, Val^67^
		O	Lys^66^	NZ	
	P3-Pro				Gln^155^, Met^156^, Tyr^159^
	P4-Pro				
	P5-Gly				
	P6-Phe				Ser^9^, Asp^69^, Asn^70^, Thr^73^, Tyr^74^, Ser^97^, Phe^99^
	P7-Glu	O	Asn^77^	ND2	Arg^114^, Trp^147^, Ala^150^, Ala^152^
	P8-Leu	O	Trp^147^	NE1	Thr^73^, Asn^77^, Trp^147^
	P9-Tyr	N	Asn^77^	OD1	Asn^77^, Ala^80^, Leu^81^, Tyr^84^,
		O	Lys^146^	NZ	Leu^95^, Asp^116^, Tyr^123^, Ile^124^,
		OH	Asp^116^	OD1	Thr^143^, Lys^146^
		OXT	Tyr^84^	OH	
			Thr^143^	OG1	
			Lys^146^	NZ	
		O	Tyr^84^	OH	
			Thr^143^	OG1	
		OXT	Tyr^84^	OH	
			Lys^146^	NZ	

The B pocket consists of Tyr^7^, Ala^24^, Val^25^, Val^34^, Met^45^, Glu^63^, Lys^66^, and Val^67^ (Figure 2B). The charged Glu^63^ and Lys^66^ residues at the top of the B pocket can form two hydrogen bonds with the main chain of P2-Met. The hydrophobic B pocket accommodates the non-polar side chain of P2-Met via the VDWs provided by the surrounding residues (Figure 2B; Table 3). The residue composition of SLA-1^*^1502 is similar to that of SLA-1^*^0401, and only the residue at position 66 (Lys/Val) is different (Figure 3).

The C, D, and E pockets usually form a large cavity in the middle portion of the PBG. The amino acid compositions of these three pockets in SLA-1^*^1502 are shown in Figures 2C–E. No hydrogen bonds or salt bridges were found in these structures; instead, many VDWs were observed between the three pockets and the NSP9-TMP9 peptide (Table 3). The D pocket is critical for the peptide selection of SLA-1^*^0401 and SLA-3^*^hs0202 because of the charged residue at position 156 (19, 20). The non-polar Met^156^ causes the D pocket of SLA-1^*^1502 to be hydrophobic, in contrast to the charged D pocket of SLA-1^*^0401 or SLA-3^*^hs0202 (Figure 3).

The F pocket of pSLA-1^*^1502 consists of Asn^77^, Ala^80^, Leu^81^, Tyr^84^, Leu^95^, Asp^116^, Tyr^123^, Ile^124^ Thr143, and Lys^146^ and shows numerous interactions with P9-Tyr, reflecting a key anchoring site (Figure 2F). P9-Tyr can form 6 hydrogen bonds and many VDWs with the residues of the F pocket (Table 3). The F pockets of both pSLA-1^*^1502 and p/SLA-1^*^0401 can accommodate P9-Tyr, and only two different residues (Asn/Gly^77^ and Ala/Thr^80^) were found between the two F pockets (Figure 3).

**Figure 4:**
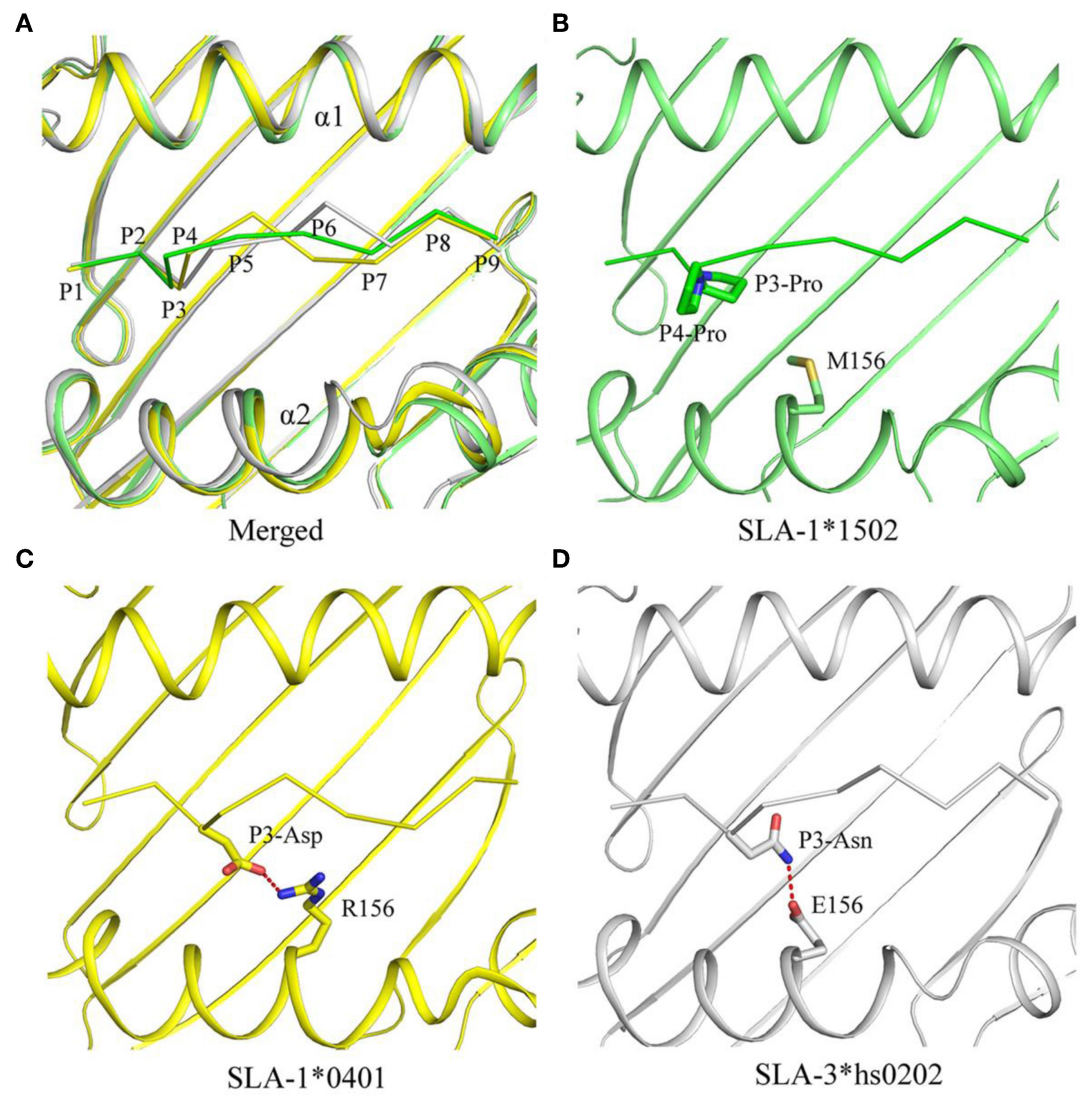
Comparison of NSP9-TMP9 and dissimilar peptide conformations and interactions among the three solved SLA I structures. The superposition of the structures of pSLA-1*1502 (green), p/SLA-1*0401 (yellow), and p/SLA-3*hs0202 (white). **(A)** The peptide conformations in these three structures are different. **(B–D)** Interactions between residue 156 and non-apeptide in the three p/SLA complexes. The salt bridge between the peptide and SLA I is represented by red dashes.

The NSP9-TMP9 peptide conformation presented by SLA-1^*^1502 is different from that of the peptides presented by SLA-1^*^0401 and SLA-3^*^hs0202 (Figure 4). Because of the two consecutive Pro residues, the turn between P3 and P4 of the NSP9-TMP9 peptide is much sharper than that in the other two peptides (Figures 4A,B). Previous studies on SLA-1^*^0401 and SLA-3^*^hs0202 showed that the residue at position 156 plays a key role in peptide binding by fixing the P3 residue with a salt bridge or hydrogen bond (Figures 4C,D). In contrast, no salt bridge or hydrogen bond forms between the P3-Pro of NSP9-TMP9 and Met^156^ of SLA-1^*^1502.

### Analysis of the Peptide-Binding Motif of pSLA-1^*^1502

**Figure 5:**
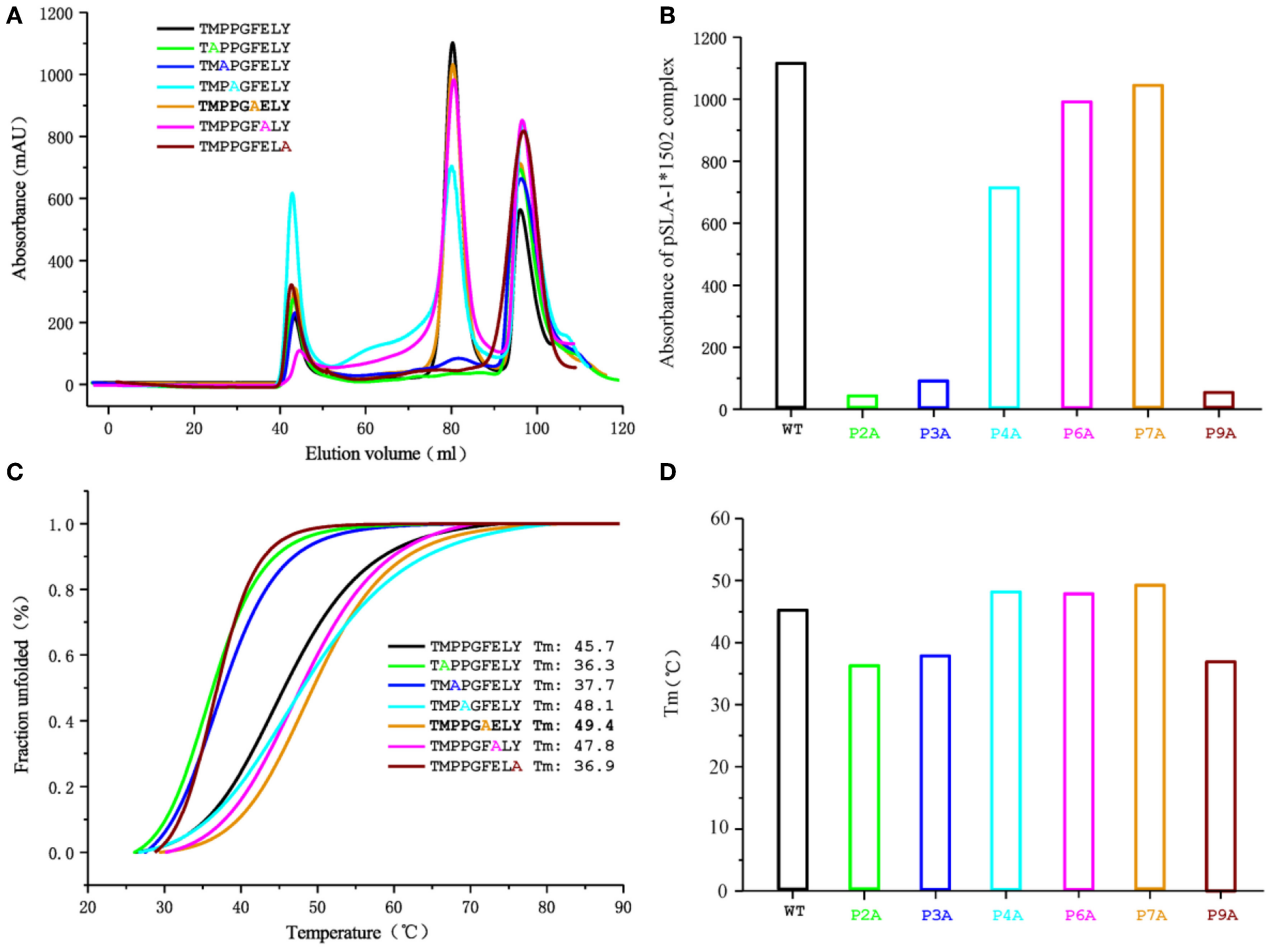
Gel filtration chromatogram and CD spectra of the NSP9-TMP9 peptide and its mutants. The elution curves and CD spectra of the complexes with different peptides are indicated in different colors. **(A)** Gel filtration chromatograms of the refolded products obtained with a Superdex 200 10/300 GL column (GE Healthcare). The aggregated H chain, the correctly refolded pSLA-1*1502 complex (~45 kDa), and excess β2m are indicated by three peaks appearing in order. The efficiency of refolding is represented by the height of the pSLA-1*1502 complex peak. A higher peak indicates better efficiency of the peptide in promoting the renaturation of SLA I. **(B)** Bar graph of the peak absorbance of the pSLA-1*1502 complex. Bars with different colors show the differences in the refolding efficiencies of the pSLA-1*1502 complex with alanine scanning peptides. **(C)** Thermostability of the pSLA-1*1502 complex with the NSP9-TMP9 peptide and the peptide harboring substitutions (alanine screening of NSP9-TMP9). CD spectra were utilized to assess the thermostability of purified pSLA-1*1502 complexes. Denaturation was monitored at 218 nm as the temperature was ramped up from 25 to 90°C at 1°C/min. The fitting data for the denaturation curves obtained using Origin 9.1 (OriginLab) are shown. The Tms of different peptides (when 50% of the fraction was unfolded) are indicated by the gray line. **(D)** Bar graph of the thermostability of the pSLA-1*1502 complex. Bars with different colors show the Tm values of the pSLA-1*1502 complex with alanine scanning peptides more clearly.

To determine the peptide-binding motif of SLA-1^*^1502, the peptide NSP9-TMP9 was mutated by alanine scanning (19), and CD spectra were used to test the stability of pSLA-1^*^1502 complexes with these mutant peptides (Figure 5). The *in vitro* refolding and CD results showed that the binding stabilities of P2-Ala, P3-Ala, and P9-Ala mutant peptides are significantly lower than that of the wild-type NSP9-TMP9 peptide. Although P3-Pro cannot form a hydrogen bond or salt bridge with the D pocket of SLA-1^*^1502, its Ala mutant still impairs the stability of the pSLA-1^*^1502 complex. According to these results, the P2, P3, and P9 residues are the primary anchor residues of the epitope peptides presented by SLA-1^*^1502. The B, D and F pockets accommodate these primary anchor residues and determine the peptide-binding motif of SLA-1^*^1502, similar to SLA-1^*^0401 and SLA-3^*^hs0202.

The B and F pockets accommodate the P2 and P9 anchor residues of the binding peptide, respectively, and their preference for P2 and P9 anchor residues is determined by their amino acid composition. Figure 2 shows the pocket composition of SLA-1^*^1502. Figure 3 shows that the amino acid composition of the B and F pockets of SLA-1^*^1502 is very similar to that of SLA-1^*^0401. Differential amino acids are found only at one or two individual sites and do not form direct contacts with the side chains of the P2 or P9 residues of the binding peptide. Because of the similar B and F pockets, the P2 and P9 residues of the SLA-1^*^1502-binding peptides should be the same as in the SLA-1^*^0401 peptides. The SLA-1^*^0401 binding peptide (20) and the SLA-1^*^1502 binding peptide have a large overlap at the P2 and P9 residues (Table 1). The B pocket of SLA-1^*^1502 accommodates multiple uncharged residues, while the F pocket mainly binds Phe, Tyr and Trp. The *in vitro* refolding results for the peptides supported this reasonable speculation (Table 1). The uncharged D pocket of SLA-1^*^1502 might accommodate various uncharged P3 residues, unlike those of SLA-1^*^0401 and SLA-3^*^hs0202. Peptides with P3-Ala cannot provide sufficient affinity, unlike larger amino acids, such as L, M, F, S, N, and P (Table 1). In summary, the preliminary peptide-binding motif of SLA-1^*^1502 is expected to contain the following combination: X-(S/M/F/W/T/V/I/L)-(L/P/M/F/S/N)-X-X-X-X-X-(F/Y/W).

### Identification of Peptide-Binding Maps for the SLA-1^*^1502 Allele Derived From Four Typical PRRSV Strains

**Figure 6:**
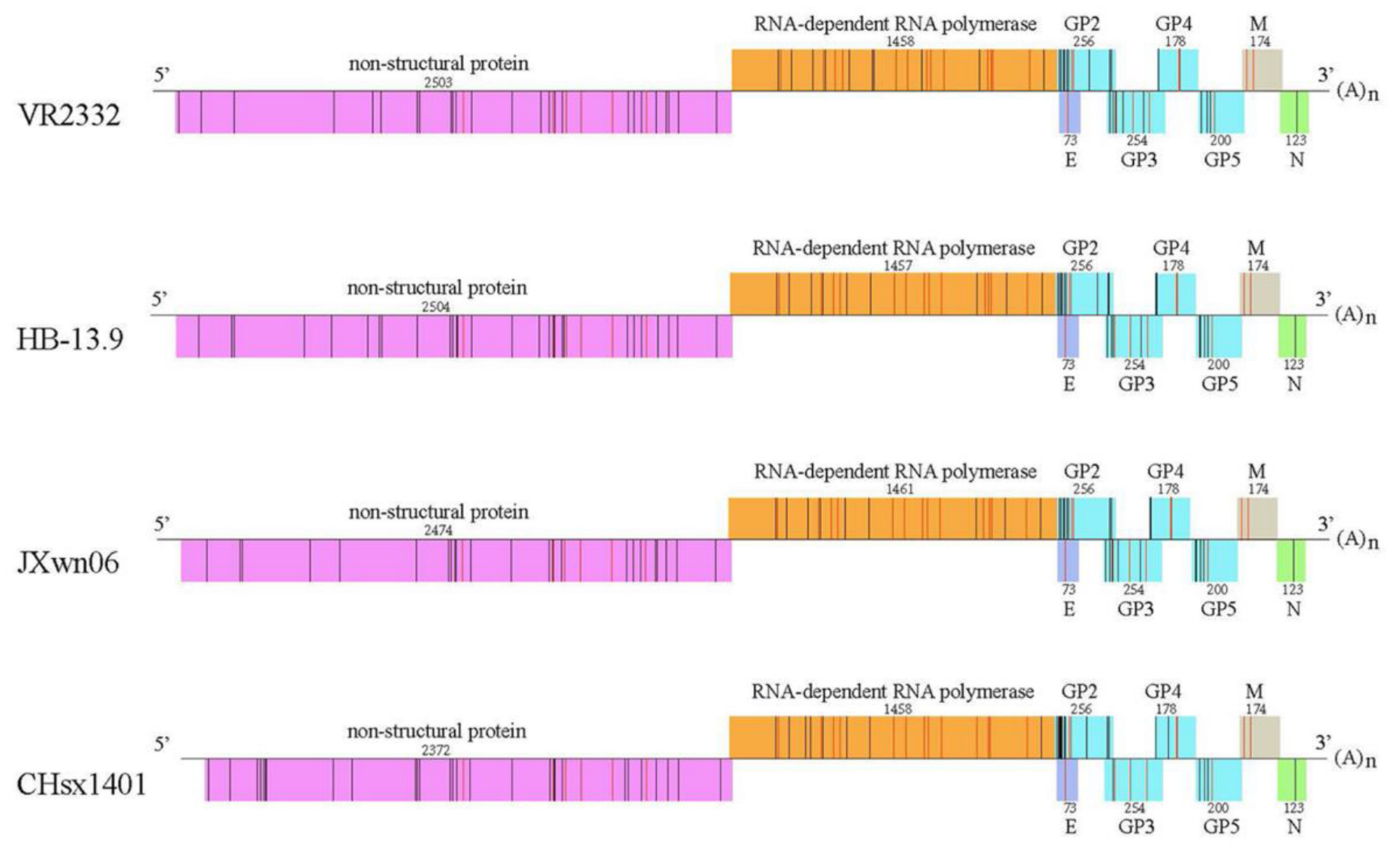
Peptide predictions from different PRRSV strains according to the binding motifs of the pSLA-1*1502 complex. Genome-wide scanning results for peptides matching the following motifs of the pSLA-1*1502 complex: X-(S/M/F/W/T/V/I/L)-(L/P/M/F/S/N)-X-X-X-X-X-(F/Y/W). Different proteins are indicated by different colors. The number above indicates the sequence length of each protein. The non-apeptides matching the motifs of the pSLA-1*1502 complex from different proteins are indicated by bars, and red indicates the conserved peptides in four PRRSV strains, including the first isolated strain VR2332 (GenBank accession # EF536003.1); the low-virulence strain HB-13.9 (GenBank accession # EU360130.1); the highly pathogenic strain JXwn06 (GenBank accession # EF641008.1); and the CHsx1401 strain, which was responsible for a recent epidemic in China (GenBank accession # KP861625.1).

The predicted peptide epitopes derived from whole protein sequences of four typical PRRSV strains were screened: the first isolated strain, VR2332; the low-virulence strain HB-13.9; the highly pathogenic strain JXwn06; and the CHsx1401 strain, which was responsible for a recent epidemic in China (Figure 6). Most SLA-1^*^1502-restricted PRRSV peptides are located in the non-structural protein and the RNA-dependent RNA polymerase (RDRP) encoded by ORF1a and 1b. Although numerous 9-mer SLA-1^*^1502-binding peptides exist in each of these four PRRSV strains (~90 peptides), only 30 peptides were found to be completely conserved in all four strains (Table S2). RDRP contains 13 conserved peptides, which is a much greater number than in the other proteins.

### Identification of NSP9-TMP9 as the CTL Epitope by Using the Tetramer Technique and the Detection of Swine IFN-γ

**Figure 7:**
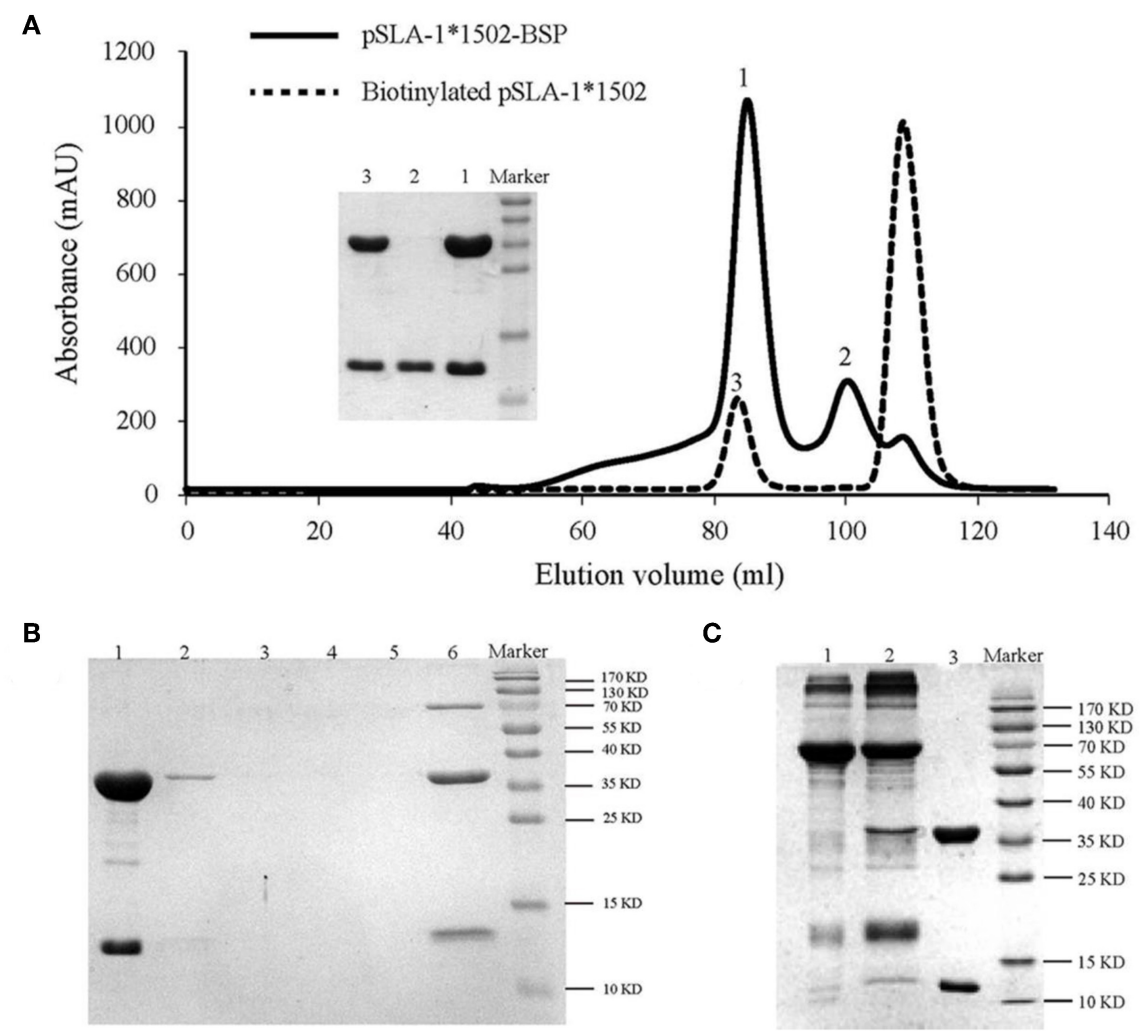
Production of the SLA−1*1502 tetramer. **(A)** The pSLA-1*1502-BSP complex with the NSP9-TMP9 peptide (solid line) and the biotinylated pSLA-1*1502-BSP complex obtained by using the BirA enzyme (dashed line) were purified via chromatography with a Superdex 200 size-exclusion column. The efficiency of purification for the complex was tested via SDS-PAGE. The pSLA-1*1502-BSP complex is shown in lane 1; sβ2m is shown in lane 2; and biotinylated pSLA-1*1502 is shown in lane 3. **(B)** SDS-PAGE analysis of the effect of pSLA-1*1502-BSP biotinylation. The biotinylated pSLA-1*1502-BSP was mixed with streptavidin MagneSpheres. Lane 1 contains the supernatant from biotinylated pSLA-1*1502-BSP that had reacted with streptavidin MagneSpheres. Lanes 2, 3, 4, and 5 contain the supernatants from the first, second, third, and fourth washings of the streptavidin MagneSpheres, respectively. Lane 6 contains the supernatant of streptavidin MagneSpheres boiled after washing the sample four times. **(C)** SDS-PAGE analysis of purified tetramers. Biotinylated pSLA-1*1502-BSP was mixed with PE-labeled streptavidin and filtered with a 100 kDa Millipore tube. Lane 1, PE-labeled streptavidin; Lane 2, pSLA-1*1502-BSP tetramer >100 kDa; and Lane 3, biotinylated pSLA-1*1502-BSP monomer.

**Figure 8:**
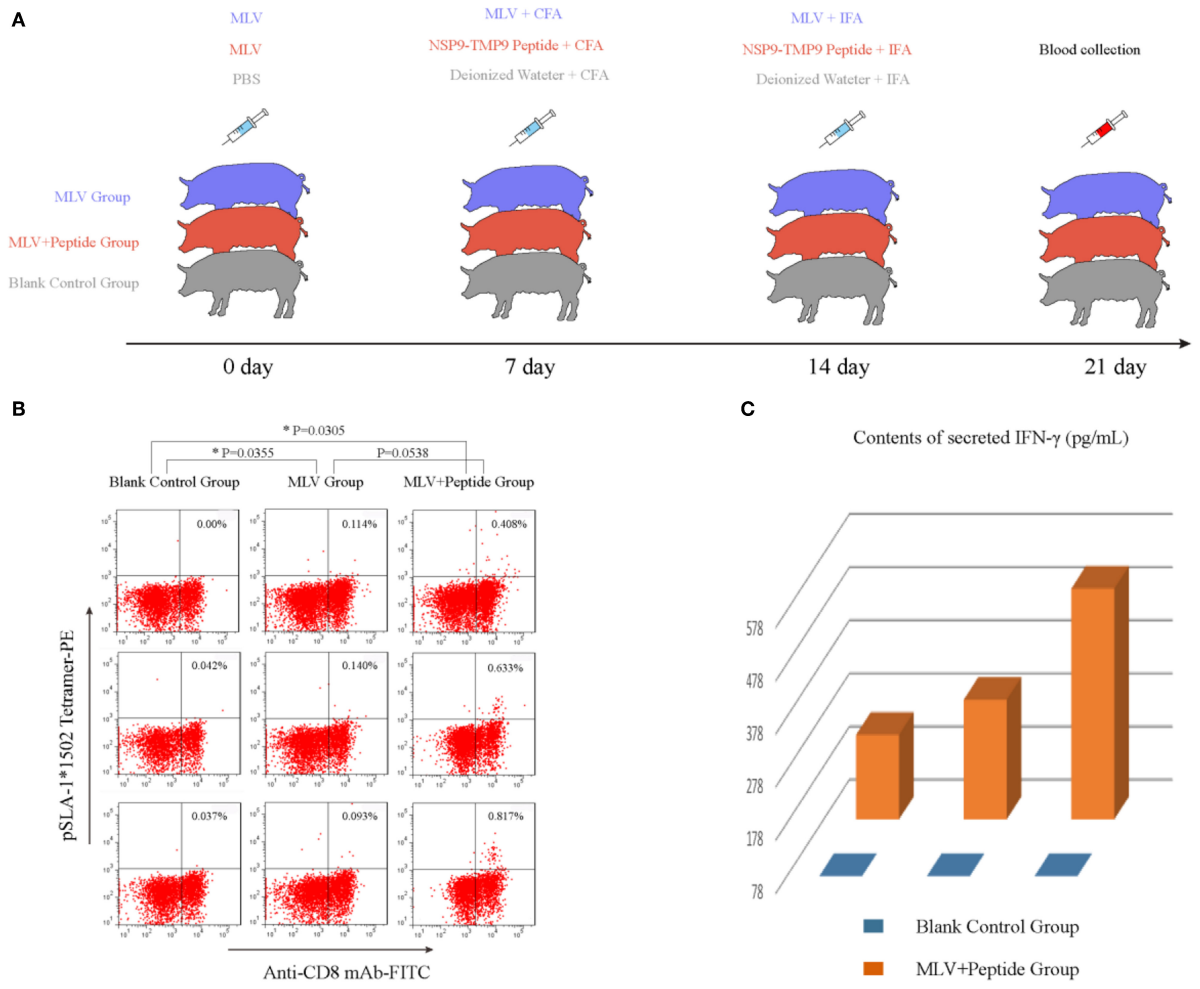
Identification of the functional NSP9-TMP9 CTL epitope. **(A)** Immunization program for SPF pigs. Nine pigs expressing SLA-1*1502 were divided into three groups: the MLV group, MLV+Peptide group and blank control group. The MLV and MLV+Peptide groups were injected with an attenuated PRRSV vaccine as the first immunization. Seven days later, the MLV+Peptide group was injected with the NSP9-TMP9 peptide mixed with CFA, and the MLV group was injected with MLV mixed with CFA as the second immunization. At day 14, the MLV+Peptide group was injected with the NSP9-TMP9 peptide mixed with IFA, and the MLV group was injected with MLV mixed with IFA as the third immunization. Pigs in control group were injected with PBS, deionized water mixed with CFA, and deionized water mixed with IFA. **(B)** NSP9-TMP9-specific CTLs stained with PE-labeled SLA-1*1502 tetramer and FITC-labeled anti-CD8 monoclonal antibody were detected via flow cytometry. **(C)** The secreted IFN-γ contents of the control group and immunized group were measured via ELISA kit. The secreted IFN-γ content of the control group was less than the lowest detectable limit (78 pg/mL).

The tetrameric pSLA-1^*^1502 complex was constructed (Figures 7A–C) (33). Six Landrace pigs expressing the SLA-1^*^1502 genes were used to check the immunogenicity of NSP9-TMP9 peptide (Figure 8). A total of 10,000 events were recorded by the flow. The ratio of pSLA-1^*^1502 tetramer and CD8 double-positive cells was at a rate of ~0.5–1% in the MLV+NSP9-TMP9-immunized group, which was significantly higher than in the control group (*P* = 0.0305). The MLV-immunized group was significantly higher than in the control group (*P* = 0.0355); however, there was no significant difference between the MLV+NSP9-TMP9-immunized group and the MLV-immunized group (*P* = 0.0538) (Figure 8B). Additionally, swine IFN-γ expression in the peripheral blood of each pig was detected according to the methods used by Kumar and Walker (34, 35). Secreted IFN-γ was detectable in all of the immunized pigs but was lower than the lowest detectable limit in all control pigs (Figure 8C). These data indicated that NSP9-TMP9, as the CTL epitope, could stimulate specific CTL immunity in swine.

## Discussion

CTL epitopes might be a requirement of optimal PRRSV immunity for the control and treatment of PRRSV infection (36, 37). In our study, a novel approach was used to select PRRSV CTL epitopes, i.e., starting from a computer prediction for PRRSV peptides, followed by *in vitro* complex refolding with SLA-1^*^1502, the analysis of the complex crystal structure, the identification of SLA-1^*^1502-restricted potential epitopes from whole genomes of different PRRSV strains, and finally, verification of the immunogenicity of SLA-1^*^1502-restricted PRRSV epitope.

The crystal structure of SLA-1^*^1502 is the third to be solved for an SLA-I allele. The crystal structure of SLA-1^*^1502 exhibits the typical structural characteristics of an MHC I complex. The structure of SLA-1^*^1502 is very similar to those of SLA-1^*^0401 and SLA-3^*^hs0202, indicating that the overall combination of heavy chains, light chains, and peptides in the swine SLA-I complex is highly conserved. However, in terms of peptide binding, the SLA-1^*^1502 structure not only reflects the common features of SLA-I alleles but also exhibits unique allelic-specific characteristics. Similar to the previously resolved SLA-1^*^0401 and SLA-3^*^hs0202, the N-terminus of the SLA-1^*^1502 PBG is open because the amino acid at position 167 of the A pocket is a small Ser (Figure 2A), but in other species such as humans and mice, the amino acid at this position is a large Trp (19, 20). The peptide-binding motif of SLA-1^*^1502, like that of SLA-1^*^0401 and SLA-3^*^hs0202, is determined by the three pockets B, D and F together, while the HLA-I molecule is mostly determined by the two pockets B and F. These common characteristics indicate that SLA-I has its own unique species features in binding peptides. In the B and F pocket composition, SLA-1^*^1502 and SLA-1^*^0401 are very similar, and only the non-critical amino acids in the individual positions are different (Figure 3), resulting in a large overlap of the anchoring residues accommodated in their B and F pockets (20). In the structures of SLA-1^*^0401 and SLA-3^*^hs0202, the D pocket plays a key role in fixing the bound peptides, with a strong salt bridge between the charged residue 156 and the P3 residue of the peptides (19, 20). The uncharged Met^156^ of SLA-1^*^ 1502 cannot form strong charge interactions with P3 residues similar to those observed for SLA-1^*^0401 and SLA-3^*^hs0202 (Figure 4). Nevertheless, the D pocket is still important in determining the peptide binding of SLA-1^*^1502 and prefers uncharged residues of a certain size to form sufficient VDWs. The three p/SLA I structures indicate that regardless of its properties, the D pocket is critical in determining the peptide-binding motif of SLA-I, and this phenomenon is expected to be a common feature among different SLA-I alleles.

SLA-1^*^1502 was predicted *in silico* to present more PRRSV peptide epitopes than other SLA-I alleles cloned from Landrace pigs, and the *in vitro* refolding results confirmed that most of the predicted PRRSV peptides could be bound by SLA-1^*^1502. Four typical PRRSV strains of the North American genotype were used to screen SLA-1^*^1502-restricted binding peptides. According to the summarized peptide-binding motifs, approximately 90 peptides in each PRRSV strain could be presented by SLA-1^*^1502. These peptides are unevenly distributed in different regions, with the NSP3/4/5 proteins encoded by ORF1a, NSP9/10/11 encoded by ORF1b and GP2/3 exhibiting most of the candidate peptide epitopes. Approximately one out of three peptides are conserved among the four PRRSV strains, and approximately half of these peptides are encoded by ORF1b of RDRP. CTL-epitope-based vaccines present advantages in terms of safety, specificity, and usability and are successfully used to control many viruses, such as HIV, HPV, and dengue virus (38–41). Although studies aimed at developing an anti-PRRSV epitope-based vaccine have been performed, no mature product is currently available (42–44). Our data indicated that RDRP (especially NSP9/10/11) may be the best target for developing a PRRSV vaccine to induce a CTL response to genetically heterologous strains.

Tetramers of p/MHC I alleles are basic reagents that are used in immunological studies (11, 45, 46). However, the absence of SLA-I tetramers limits effective and convincing research on swine antiviral CTL responses, especially regarding accurate quantitative research. In this study, the crystallized NSP9-TMP9 peptide was used to produce the tetramer for evaluating SLA-1^*^1502-restricted CTL responses. The NSP9-TMP9 epitope could induce CD8 and tetramer double-positive CTLs at a rate of ~0.5–1% in MLV+NSP-TMP9-immunized pigs, similar to the results obtained for other known efficiently protective viral CTL epitopes found in humans and mice by FACS (47–49). Our results also showed that the MLV used (produced by the VR2332 strain) could induce a CTL response specific to PRRS. Somewhat disappointingly, we do not have live PRRSV with which to challenge these swine groups and evaluate the protection of MLV and NSP-TMP9 epitope. However, NSP9-TMP9 was identified as an immunogenic epitope that could stimulate the proliferation of specific CD8^+^ CTLs and the expression of IFN-γ in SPF Landrace pigs bearing SLA-1^*^1502 alleles. Immunization enhancement with NSP9-TMP9 produces a specific CTL response similar to that of immunization with MLV, indicating that the peptide vaccine can produce effective immunoprotection and thus that it is feasible to develop an effective PRRSV polypeptide epitope vaccine.

In conclusion, we solved the crystal structure of SLA-1^*^1502 and described its PRRSV peptide-binding map according to its preliminary peptide-binding motif determined via biochemical analyses. Using the tetramer of SLA-1^*^1502, the immunogenicity of NSP9-TMP9 was identified by FACS and the expression of IFN-γ. The results increase our understanding of how to acquire a viral CTL vaccine against swine PRRS disease. In addition, this study provides a complete and credible method for identifying SLA-I-restricted viral epitopes, demonstrating the feasibility of peptide vaccines in antiviral immunity of swine. Based on our experimental results, we encourage and promote the development of a safe peptide vaccine that can effectively activate CTL immune protection, solve the safety problems caused by conventional attenuated vaccines, and provide new ideas for controlling not only PRRS but also the extent of African swine fever.

## Data Availability Statement

The coordinates and structural characteristics of pSLA-1^*^1502 have been deposited in the Protein Data Bank under accession number 5YLX; the sequence of SLA-1^*^1502 is available at the National Center for Biotechnology Information (NCBI) database under accession number HQ909439.

## Ethics Statement

The animal trials in this study were performed according to the Chinese Regulations for Laboratory Animals-The Guidelines for the Care of Laboratory Animals (Ministry of Science and Technology of People's Republic of China) and Laboratory Animal Requirements for Environment and Housing Facilities (GB14925-2010, National Laboratory Animal Standardization Technical Committee). The license number associated with this research protocol is CAU20140305-2, which was approved by The Laboratory Animal Ethics Committee of China Agricultural University. The protocol adhered to the recommendations in the Institute for Laboratory Animal Research's Guide for the Care and Use of Laboratory Animals.

## Author Contributions

CX: design of the study. XP and NZ: data collection. XP, YJ, and QL: analysis and interpretation of data. CX, NZ, and XW: drafting the article. CX, XP, RL, LZ, and LM: critical revision of the article. XP, NZ, XW, YJ, RC, QL, RL, LZ, LM, and CX: final approval of the version to be published.

### Conflict of Interest

The authors declare that the research was conducted in the absence of any commercial or financial relationships that could be construed as a potential conflict of interest.
