# Supplementary material for: Illumination of PRRSV Cytotoxic T Lymphocyte Epitopes by the Three-Dimensional Structure and Peptidome of Swine Lymphocyte Antigen Class I (SLA-I)
Source: Front Immunol. 2020 Jan 8;10:2995. doi: 10.3389/fimmu.2019.02995 (PMC6960135; doi:10.3389/fimmu.2019.02995)
Supplement: Supplementary file 1 [file Table_1.DOCX]

**Table S1.** Prediction of the potential binding peptides from PRRSV for 6 SLA-I alleles of Landrace pigs.

| Genes | Affinity(1-log50k) | | | |
| --- | --- | --- | --- | --- |
|  | 0.2-0.3 | 0.3-0.4 | 0.4-0.5 | ≥0.5 |
| LI0101(SLA-1*1502) | 76 | 29 | 8 | 3 |
| LI0102 | 3 | 0 | 0 | 0 |
| LI0201 | 3 | 0 | 0 | 0 |
| LI0202 | 0 | 0 | 0 | 0 |
| LI0301 | 0 | 0 | 0 | 0 |
| LI0303 | 0 | 0 | 0 | 0 |

PRRSV type, PRRSV-VR2332 strain (GenBank accession No. AY150564). The numbers represent the nonapeptides predicted from PRRSV for SLA-I alleles. The estimated binding affinity to SLA-I was calculated by the NetMHCpan 4.0 server (http://www.cbs.dtu.dk/services/NetMHCpan/).

**Table S2.** Peptide predictions for different PRRSVs according to the binding motifs of the pSLA-1*1502 complex.

Genome-wide scanning results for peptides matching the following motifs of the pSLA-1*1502 complex: X-(S/M/F/W/T/V/I/L)-(L/P/M/F/S/N)-X-X-X-X-X-(F/Y/W). Different colors indicate different proteins. The nonapeptides matching the motifs of the pSLA-1*1502 complex were identified, and the red peptides are the conserved peptides in these four PRRSV strains. The numbers after the peptides are the positions of the different peptides.
